# Patients with fibromyalgia display less functional connectivity in the brain’s pain inhibitory network

**DOI:** 10.1186/1744-8069-8-32

**Published:** 2012-04-26

**Authors:** Karin B Jensen, Rita Loitoile, Eva Kosek, Frank Petzke, Serena Carville, Peter Fransson, Hanke Marcus, Steven CR Williams, Ernest Choy, Yves Mainguy, Olivier Vitton, Richard H Gracely, Randy Gollub, Martin Ingvar, Jian Kong

**Affiliations:** 1 Department of Psychiatry, Massachusetts General Hospital, Harvard Medical School, Boston, USA; 2 Athinoula A. Martinos Center for Biomedical Imaging, Boston, USA; 3 Department of Clinical Neuroscience, Karolinska Institutet, Stockholm, Sweden; 4 Osher Center for Integrative Medicine, Karolinska Institutet, Stockholm, Sweden; 5 Pain clinic, Centre for Anesthesiology, Emergency and Intensive care Medicine, University Medical Centre, Göttingen, Germany; 6 UK Age Research Forum, London, UK; 7 Department of Anesthesiology and Postoperative Intensive Care Medicine, University Hospital of Cologne, Cologne, Germany; 8 Centre for Neuroimaging Science, Institute of Psychiatry, King’s College London, London, UK; 9 Department of Medicine, Cardiff University School of Medicine, Cardiff, UK; 10 Pierre Fabre Médicament, Labège, France; 11 Center for Neurosensory Disorders, University of North Carolina, Chapel Hill, USA

## Abstract

**Background:**

There is evidence for augmented processing of pain and impaired endogenous pain inhibition in Fibromyalgia syndrome (FM). In order to fully understand the mechanisms involved in FM pathology, there is a need for closer investigation of endogenous pain modulation. In the present study, we compared the functional connectivity of the descending pain inhibitory network in age-matched FM patients and healthy controls (HC).

We performed functional magnetic resonance imaging (fMRI) in 42 subjects; 14 healthy and 28 age-matched FM patients (2 patients per HC), during randomly presented, subjectively calibrated pressure pain stimuli. A seed-based functional connectivity analysis of brain activity was performed. The seed coordinates were based on the findings from our previous study, comparing the fMRI signal during calibrated pressure pain in FM and HC: the rostral anterior cingulate cortex (rACC) and thalamus.

**Results:**

FM patients required significantly less pressure (kPa) to reach calibrated pain at 50 mm on a 0–100 visual analogue scale (*p* < .001, two-tailed). During fMRI scanning, the rACC displayed significantly higher connectivity to the amygdala, hippocampus, and brainstem in healthy controls, compared to FM patients. There were no regions where FM patients showed higher rACC connectivity. Thalamus showed significantly higher connectivity to the orbitofrontal cortex in healthy controls but no regions showed higher thalamic connectivity in FM patients.

**Conclusion:**

Patients with FM displayed less connectivity within the brain’s pain inhibitory network during calibrated pressure pain, compared to healthy controls. The present study provides brain-imaging evidence on how brain regions involved in homeostatic control of pain are less connected in FM patients. It is possible that the dysfunction of the descending pain modulatory network plays an important role in maintenance of FM pain and our results may translate into clinical implications by using the functional connectivity of the pain modulatory network as an objective measure of pain dysregulation.

## Introduction

FM is a common pain disorder, estimated to affect 2 to 4 % of the population, out of which 80 % are women [1]. This multi-symptomatic pain syndrome is characterized by widespread musculoskeletal pain, stiffness, soft tissue tenderness, general fatigue and sleep disturbances [2]. Effective treatment for FM is scarce and long-term follow-up demonstrates that FM is chronic, with recurrent periods of intensified symptoms and low probability of full recovery [3]. As a result, FM often has devastating effects on quality of life, including lost productivity and increased healthcare costs [4-6]. The strongest precursor to FM is the presence of long-term localized pain [7], suggesting that FM pathology develops over time in exposure to persistent pain. A better understanding of central pain modulation in FM, and therefore of its underlying mechanisms, could help to develop strategies aimed at prevention and treatment.

Accumulated evidence indicates that pain dysregulation within the central nervous system plays an important role in the development and maintenance of FM. For instance, brain imaging studies suggest that FM is associated with 1) augmented brain responses to experimental pain stimuli [8,9]; 2) changed resting-state functional connectivity [10]; 3) changes in brain morphology (atrophy) in brain regions implicated in pain processing [11-13] and 4) altered function of brain neurotransmission [14-17]. A number of behavioral studies have suggested that the Descending Pain Modulatory System (DPMS) is impaired in FM [18-21]. However, the DPMS hypothesis has been poorly explored in brain imaging studies. In a previous study, we provided indirect evidence for DPMS dysfunction in FM by comparing pain-evoked brain activity in FM patients and healthy controls during conventional fMRI. Results showed that FM patients failed to activate the rostral anterior cingulate cortex (rACC) - a key region for the descending inhibition of pain [22]. In line with findings from previous neuroimaging studies [9,23,24], we also found decreased thalamic activation, indicating altered regulation of incoming pain signals in FM.

Recently, several fMRI studies have significantly enhanced our understanding of the intrinsic functional connectivity underlying pain perception [25,26], pain modulation [27] and pain pathology [28,29]. In a previous study [27], we found that key regions for descending pain modulation, including the rACC, PAG and Rostral Ventromedial Medulla, were functionally connected during resting state in healthy individuals; indicating that functional connectivity could be a useful tool to investigate the pain modulatory system.

In this study we used connectivity analyses of fMRI data to investigate the functional connectivity of the rACC and thalamus during pressure-evoked pain, in both healthy controls and FM patients. Previous neuroimaging studies in FM found altered cerebral activity during evoked pain [8,9,22], suggesting that the functional connectivity during active processing of pain may better represent the pathophysiology of FM. We hypothesized that, compared to healthy controls, FM patients would display less pain-evoked functional connectivity between our seed regions (rACC and thalamus) and other nodes in the pain modulatory system.

## Material and Methods

### Participants

A total of 42 female subjects (28 FM patients and 14 healthy controls) were analyzed in the present study. Patients were recruited as part of a pharmacological multicenter study (EudraCT # 2004-004249-16). All three sites – one in England, Sweden and Germany, respectively - also performed the experiment in healthy controls, consecutively recruited throughout the study. A total of 92 female FM patients aged 25 to 55 years, mean age 44 (SD = 8.2) were enrolled. Of the 92 patients 9 were excluded from fMRI analyses due to image artifacts or *en passant* findings of intracranial anomalies, leaving 83 patients for matching with healthy controls. A total of 14 healthy female controls were available, aged 24 to 48 years, mean age 34 (SD = 8.6). Due to the relatively large number of patients, each healthy control could be age-matched with two FM patients (n = 28, range 24–48 years, mean 38, SD = 6.8). In cases where there were more than two patients with the same age, the two patients with the most similar duration of FM symptoms were chosen. The study was performed in accordance with the Helsinki Declaration and was approved by the local Ethical Committee at each of the three participating sites. All patients and healthy controls gave written informed consent. Parts of the present dataset were used in 2 previous publications; see Jensen et al. 2010 [30] where all 92 available FM patients were analyzed, and Jensen et al. 2009 [22], where 11 FM patients and 14 controls from the present study were included.

### Procedure

All subjects were first screened and then scheduled for one behavioral session and one fMRI session on two adjacent days. On day one, subjects were familiarized with the equipment that was used to evoke experimental pain, calibrated for subjective pain ratings and asked to fill out self-report questionnaires. FMRI scanning was performed on day two.

### Screening

All subjects were right-handed. The study protocol required that all medications that could influence pain perception, including psychopharmacological medications, were washed out. Patients had to withdraw from all central nervous system acting therapies, including antidepressants, anticonvulsants, mood stabilizers, opioids, and narcotic patches, and to discontinue treatment with transcutaneous electrical nerve stimulation, biofeedback, tender and trigger point injections, acupuncture, and anesthetics. Required periods off medication were dependent on the actual pharmacological characteristics to ensure complete washout. All analgesics were prohibited during the study, except for paracetamol, dipyrone and nonsteroidal anti-inflammatory agents (NSAIDs), which were used as rescue medications. The rescue medications had to be prescribed at the lowest available dose and for the shortest period of time necessary to manage the patient’s acute pain. Use of any analgesic or narcotic drug had to be discontinued 48 hours prior to the assessments of pain sensitivity. Zolpidem was allowed for treatment of insomnia. Exclusion criteria included the following: severe psychiatric illness (including severe melancholic depressive episode); serious suicide risk; history or behavior that would prohibit study compliance; history of substance, drug, or alcohol abuse; heavy cigarette smoking (> 25 cigarettes/day); presentation of an intracranial anomaly; significant cardiovascular, pulmonary, gastrointestinal, hepatic, or renal disease; history of autoimmune disease; current systemic infection; active cancer (except basal cell carcinoma) or current cancer therapy; unstable endocrine disease; severe sleep apnea; or pregnancy or breastfeeding. FM patients were mainly recruited from primary care and were diagnosed according to the 1990 American College of Rheumatology criteria [2]. All patients had a self-reported average pain intensity of at least 40 mm on a 100 mm visual analogue scale (VAS) over the previous week. For healthy controls, the exclusion criteria included presence and/or history of any clinical pain problem.

### Pain stimulation

Calibration of pain and stimulations during fMRI were performed using a laptop-controlled tool that delivered pressure to the right thumbnail. This specially designed tool, which consists of a plastic piston that applies pressure via a 1 cm^2^ hard rubber probe, has been proven effective in several previous publications [9,31,32]. Calibration was performed one day before fMRI scanning. Subjects were instructed to rate the intensity of the pain evoked by each stimulus by putting a mark on a 0–100 mm horizontal VAS ranging from “no pain” to “worst imaginable pain”. Each subject’s pain threshold and tolerance were determined in an ascending series of pressures, using 50 kPa increments (tolerance was determined as the first pressure where the patient rated > 60 mm VAS). These values were then used to create a randomized series of five different pressure intensities within the range of each subject’s threshold and tolerance. For example, if the pain threshold was represented by 200 kPa, and the maximum pain rating by 600 kPa, the randomized series would consist of pressures of 200 kPa, 300 kPa, 400 kPa, 500 kPa and 600 kPa. In total, 15 stimuli were delivered in a randomized order at 30 seconds intervals. The duration of each pressure stimulus was 2.5 seconds. Pain ratings from the randomized series were used to calculate each subject’s calibrated pressure at 50 mm VAS. This calibrated pressure was used during fMRI scanning on the following day.

### Imaging with fMRI

Images were collected using three different 1.5 Tesla scanners: in London a General Electric HDx scanner was used, in Stockholm a General Electric Twinspeed Signa Horizon, and in Cologne a PHILIPS scanner was used. Multiple T2*-weighted single-shot gradient echo EPI sequences were used to acquire blood oxygen level dependent (BOLD) contrast images. The following parameters were used: repetition time: 3000 ms (35 slices acquired), echo time: 40 ms, flip angle: 90 degrees, field of view: 24 × 24 cm, 64 × 64 matrix, 4 mm slice thickness with 0.4 mm gap and sequential image acquisition order. In the scanner, cushions and headphones were used to reduce head movement and dampen scanner noise. Placing a blank screen in front of the patient’s field of view from inside the scanner minimized visual distraction during scans.

Two types of stimulations were used during the functional scans: individually calibrated painful pressure, representing each subject’s 50 mm VAS, and a non painful pressure perceived as light touch, representing 0 mm VAS. All stimulations were randomly jittered over the scanning time, preventing subjects from anticipating the onset time and event type. The time interval between consecutive events was randomized with a mean stimulus onset asynchronicity (SOA) of 15 seconds (range 10–20 seconds). The total duration of the scans was approximately 35 minutes. Before scanning, subjects were instructed to focus on the pressures on the thumb and not to use any distraction or coping techniques.

In addition to the functional scans, high-resolution T1-weighted structural images were acquired in coronal orientation for anatomical reference purposes and screening for cerebral anomalies. Parameters were: Spoiled Gradient Recalled 3D sequence, repetition time: 24 ms, echo time: 6 ms, flip angle 35 degrees with a voxel size of 0.9 × 1.5 × 0.9 mm^3^.

The scanning procedure was standardized between sites by the use of written manuscripts for the oral instructions as well as practical training for all investigators involved in the study. In order to ensure calibrated experimental procedures and scanner settings, several visits were done at the three sites by a central coordinator.

### Self-report measures

The day before fMRI-scanning, all patients used a 0–100 mm VAS to rate their average clinical pain during the previous week and their current pain. Additionally, patients completed the Fibromyalgia Impact Questionnaire (FIQ); a 20-question questionnaire that assesses the overall symptom severity in patients with FM [33]. A high score on the FIQ corresponds to high self-reported FM severity.

### Analysis of behavioral data

The differences between FM patients and healthy controls regarding age and pressure pain sensitivity were assessed using two independent samples t-tests (two-tailed). A polynomial regression was used to determine each individual’s representation of VAS 50 mm, built on the 15 ratings from the randomized series of thumb pressures. The SPSS statistics software, version 18, was used for statistical analyses of behavioral data.

### Functional connectivity analysis

The connectivity fMRI analyses were performed in line with the methods previously described by our group [26,27,34] and other groups [35,36]. In brief, functional data was preprocessed to decrease image artifacts, between-slice timing differences, and differences in odd/even slice intensity. The data was then spatially smoothed, using a Gaussian kernel of 6 mm full-width at half-maximum and temporally filtered (0.009 Hz < f < 0.08 Hz). Several spurious or nonspecific sources of variance were removed by including the following variables in the overall statistical model: 1) six movement parameters computed by rigid body translation and rotation during preprocessing, 2) mean whole brain signal, 3) mean brain signal within the lateral ventricles, and 4) the mean signal within a deep white matter ROI. Inclusion of the first temporal derivatives of these regressors within the linear model accounted for the time-shifted versions of spurious variance. The functional connectivity analysis produced coefficients for each seed-to-voxel correlation, and Fisher’s r-to-z transformation was used to convert these correlation maps into z maps. Group effects were tested with random-effects analyses using Matlab 7 (Mathworks) and the Statistical Parametric Mapping 5 software (SPM5) (http://www.fil.ion.ucl.ac.uk/spm/). Two separate one-sample t-tests were performed for the rACC and thalamus, assessing the connectivity between the seeds and the rest of the brain in FM patients (n = 28) and healthy controls (n = 14), respectively. Differences in rACC and thalamus connectivity between FM patients and healthy controls were assessed by two separate two-sample t-tests. The two seeds used in this study were 1) the rACC, centered at x = 8, y = 46, z = 4 (Montreal Neurological Institute [MNI] coordinates) with a 2 mm radius, and 2) the thalamus, centered at x = −14, y = −34, z = 12 MNI with a 2 mm radius. The coordinates were based on the results from a previous publication where we compared the pain-evoked fMRI signal in FM patients and healthy controls [22].

According to our hypothesis and previous studies of the DPMS [37], the PAG/brain stem [38], amygdala [39], nucleus accumbens [40], hippocampus [41], and prefrontal cortex [42] were defined as regions of interest. For predefined anatomical regions of interest (ROI’s), the statistical threshold was set at voxel-wise *p* < .005, uncorrected, with a minimum cluster size of 20 contiguous voxels. For all other brain regions, a threshold of voxel-wise *p* < .05, corrected for multiple comparisons, was used.

## Results

### Behavioral results

**Figure 1:**
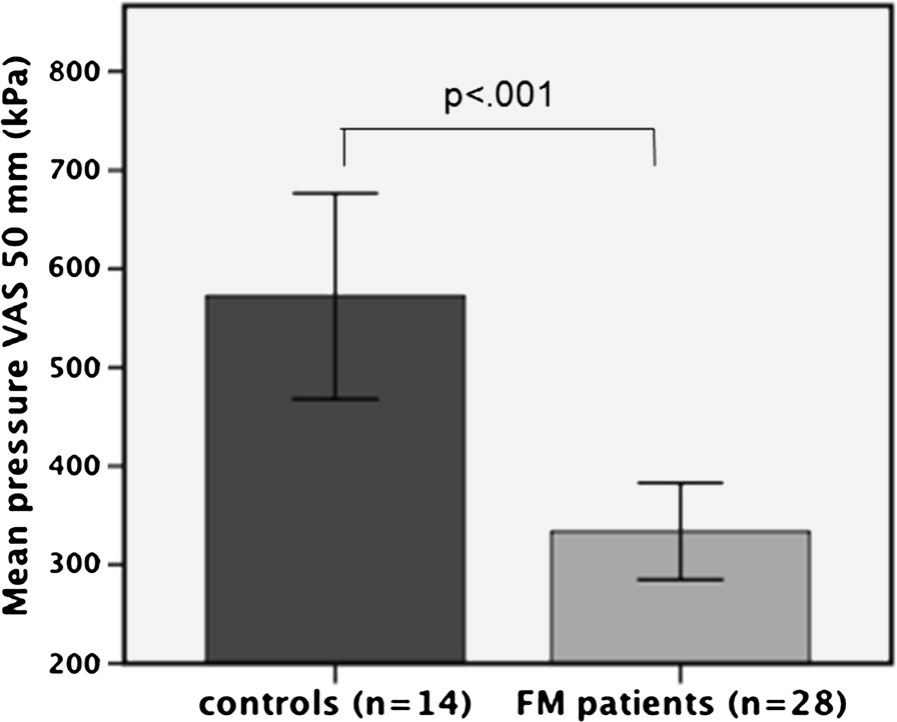
**Pressure needed to evoke the same subjective levels of pain in FM patients and controls.** A total number of 15 thumb-pressures were given in order to calculate the pressure that would represent a pain intensity of 50 mm VAS (0–100). A two-samples *t*-test confirmed that patients required significantly less pressure (kPa) than controls in order to reach VAS 50 mm; *p* < .001, two-tailed.

Each healthy control subject (n = 14) was age-matched with two FM patients (n = 28). A two-sample *t*-test validated that there was no significant difference in age between the two groups (t = −1.59, *p* = 0.126). In accordance with our previous findings, the sensitivity to pressure was significantly higher in FM patients, represented by lower amounts of pressure required to evoke pain at 50 mm VAS (t = 4.14, *p* < .001) (see Figure 1).

### Neuroimaging results

#### rACC functional connectivity

**Figure 2:**
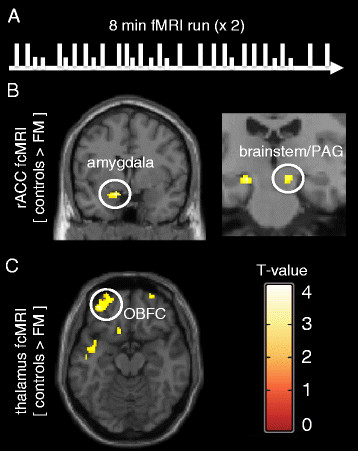
**Experimental pain paradigm and functional connectivity results.** The schematic illustration (**A**) represents the experimental pressure pain paradigm used during fMRI scanning. Calibrated painful pressures (long lines), representing 50 mm VAS, and non-painful pressures (short lines) were randomly delivered to the thumbnail during 2 adjacent 8 minute runs with 20 painful and 10 non-painful stimuli in each run. Functional connectivity results for the rACC seed (**B**) revealed incraesed connectivity to the amygdala (peak coordinate x = −14, y = 0, = − 16) and a cluster encompassing the brainstem/PAG/hippocampus (peak coordinate x = 12, y = 24, z = −12) in healthy controls, comapred to FM patients. Functional connectivity results for the thalmaus seed (**C**) revealed increased connectivity to the OBFC (peak coordinate x = −34, y = 50, z = −18) in healthy controls, compared to FM patients. All anatomical locations are given in Montreal Neurological Institute coordinates (MNI).

A one-sample *t*-test for FM patients only (n = 28), demonstrated significant functional connectivity between the seed located in the rACC and the contralateral rACC and medial prefrontal cortex (MPFC), an area which was predefined as an ROI. Also, there was significant connectivity between the rACC and the precuneus and occipital cortex (for all results see Table 1; ROI results indicated in italics). A one-sample *t*-test for healthy controls demonstrated similar functional connectivity between the seed region in the rACC and the MPFC, precuneus and the parietal cortex. A direct comparison between the healthy controls and FM patients (two sample *t*-test) revealed that, compared to FM patients, the rACC of healthy controls had significantly higher connectivity to the left amygdala, bilateral hippocampi, and the brainstem (Figure 2). There were no regions where FM patients showed higher rACC connectivity than the controls. For all rACC connectivity results, see Table 2; ROI results indicated in italics. In an exploratory analysis of our data using a lower threshold for continuous voxels (5 voxels instead of 20 voxels), we found that the RVM, a key region receiving projections from the PAG, showed higher rACC connectivity in healthy controls, compared to FM patients (RVM, *p* < .005, t = 3.13, 8 voxels, MNI coordinates: x = −2, y = −18, z = −38).

**Table 1:** Subjects’ characteristics

**Variable**	**FM patients** **(n = 28)**	**Controls** **(n = 14)**	**t-score**	**p-value**
Age (years)	37.8 (6.8)	33.6 (8.6)	−1.59	0.126
Pressure at VAS 50 mm (kPa)	333.7 (129.8)	572.4 (195.1)	4.14	0.001*
Duration of FM symptoms (months)	123.8 (76.8)	Na		
Weekly pain intensity (VAS mm)	72.3 (13.3)	Na		
Current pain intensity (VAS mm)	61.4 (22.1)	Na		
FIQ score	71.0 (12.6)	Na		

*significant at *p* < .001.

The mean value and Standard Deviation (SD) is given for each variable. For the variables ‘Age’ and ‘Pressure at VAS 50 mm’, the t-score and p-value from an independent samples *t*-test is also listed.

**Table 2:** Functional connectivity results in patients with FM and healthy controls

**rACC seed: FM patients only (n = 28)** **(MNI: x = 8, y = 46, z = 4)**	**Maximum voxel**	**Cluster** **size**	**Maximum** **t-score**	**Cluster** **p-value**
*rACC/Medial prefrontal cortex*	−8, 46, 4	16513	33.65	0.000**
Precuneus	6, −60, 26	2719	5.45	0.000**
Occipital cortex	44, −74, 32	611	4.78	0.000**
Occipital cortex	−44, −72, 38	516	4.39	0.001**
**rACC seed: Healthy controls only (n = 14)**				
*rACC/Medial prefrontal cortex*	−8, 46, 4	7122	22.69	0.000**
Posterior Cingulum/Precuneus	2, −40, 38	4180	6.40	0.000**
Parietal/Angular cortex	−56, −60, 22	1020	5.62	0.000**
Parietal/Angular cortex	46, −70, 30	693	5.08	0.000**
**rACC seed: FM patients > Healthy controls**				
No significant regions				
**rACC seed: Healthy controls > FM patients**				
*Amygdala/Parahippocampal*	−14, 0, −16	60	4.33	0.007*
*Hippocampus*	−20, −24, -12	51	4.23	0.009*
*Brainstem/PAG/Hippocampus*	12, -24, -12	32	3.61	0.018*
**Thalamus seed: FM patients only (n = 28)** **(MNI: x = −14, y = −34, z = 12)**				
*Thalamus*	14, −34, 12	5523	30.91	0.000*
**Thalamus seed: Healthy controls only (n = 14)**				
*Thalamus*	14, −34, 12	989	21.81	0.000**
*Orbitofrontal cortex*	−34, 50, −18	220	4.49	0.047**
**Thalamus seed: FM patients > Healthy controls**				
No significant regions				
**Thalamus seed: Healthy controls > FM patients**				
*Orbitofrontal cortex*	−22, 54, −17	50	3.79	0.008*

** whole brain corrected for multiple comparisons * cluster corrected for multiple comparisons.

Brain regions exhibiting significant functional connectivity with the seed regions rACC and thalamus, respectively, during calibrated pressure-evoked pain (n = 42). The seed regions, together with the PAG, amygdala, Nucleus Accumbens, hippocampus and prefrontal cortex, were considered regions of interest (ROI’s) and are marked in italics. The exact anatomical locations (x, y, z) are given in MNI coordinates. All results are corrected for multiple comparisons.

#### Thalamic functional connectivity

A one-sample *t*-test for FM patients only, demonstrated significant functional connectivity between the seed located in the thalamus and the contralateral thalamus. A one-sample *t*-test for healthy controls revealed significant functional connectivity between the seed region in the thalamus and the contralateral thalamus as well as the orbitofrontal cortex (OBFC). In a direct comparison between healthy controls and FM patients (two sample *t*-test), the thalamus showed significantly higher connectivity to the OBFC in healthy controls. There were no significant regions outside of the predefined ROI’s and no regions showed higher thalamus connectivity in FM patients, compared to controls. For all thalamic connectivity results, see Table 2.

## Discussion

In this study, we investigated the differences in functional brain connectivity between FM patients and matched healthy controls during intermittent application of pressure pain. The results revealed that healthy controls had higher rACC connectivity to the bilateral hippocampi, amygdala, brainstem and RVM; regions highly involved in the descending modulation of pain. Additionally, we found that healthy controls displayed higher connectivity between the thalamus and the OBFC, a cortical region that plays an important role in endogenous modulation of pain and emotions [43]. Hence, the present neuroimaging study provides evidence for lower functional connectivity within the pain inhibitory network of the brain in patients with FM.

### rACC connectivity

In a previous study, we found that patients with FM failed to recruit the rACC during evoked pressure pain [22]. Using the same region as seed for the present connectivity fMRI analysis, we found that healthy controls displayed higher rACC connectivity than FM patients to the hippocampus, a cluster that extended into the PAG area of the brainstem. It is well known that descending inhibitory pathways in the brain play a crucial role in pain modulation. The PAG receives direct projections from regions within the limbic forebrain such as ACC and the amygdala [37,39,44,45] and can modulate pain perception through brainstem structures, such as the Rostral Ventromedial Medulla (RVM), that directly communicate with nociceptive neurons in the dorsal horn of the spinal cord [37,45]. In an exploratory analysis of our data, using a lower threshold for continuous voxels, we found that the RVM had higher rACC connectivity in healthy controls, compared to FM. Given the crucial role of the rACC-PAG-RVM network in pain inhibition [38], our findings provide neuroimaging evidence for dysfunction of descending pain inhibition in FM. It is important to note that some brain structures, such as the ACC, may be divided in sub-regions that serve different aspects of pain processing [46], and therefore a region can sometimes be associated with increases or decreases in response to pain modulation [47], depending on the exact anatomical location and experimental paradigm. Recently published animal studies found augmented synaptic transmission in the ACC in response to long-term exposure to peripheral pain [48] and there is evidence suggesting that the rACC is required for the reward associated with pain relief [49]. Activation of the rostral ACC in humans has been demonstrated during inhibition of pain [50,51], whereas activation of the more posterior parts of the ACC has been associated with increased pain affect [52-54] in humans.

The role of the hippocampus in pain modulation is less explored but we speculate that the connectivity between the rACC and the hippocampus is likely related to the aversive drive and motivational dimension of pain [41,55]. For example, increased activation of the hippocampus has been reported in response to anxiety-induced exacerbation of pain [41] and nocebo-induced hyperalgesia [56]. It is possible that the relatively lower connectivity between the rACC and the hippocampus in FM patients represents an attenuated defensive response to pain. Similar to reports of lower opioid binding potentials in FM [14], it is possible that the constantly ongoing nociceptive activity in patients with FM causes a ceiling effect for homeostatic pain control, expressed as relatively lower connectivity between pain defensive regions like the rACC and the hippocampus, in response to experimental pain.

In addition to the functional connectivity between the rACC, hippocampus and PAG, control subjects showed significantly higher connectivity between the rACC and bilateral amygdalae, a limbic structure with direct projections to the PAG [37]. Previous studies found that the amygdala is highly involved in opioid-dependent analgesia and has been described as a crucial subcortical node for endogenous pain inhibition [57]. In line with the proposed role of the hippocampus, the activation of amygdala in relation to pain is proposed to represent a defensive mechanism that contributes to the recruitment of descending inhibition [50]. Thus, the reduced connectivity between the rACC and amygdala in FM patients may indicate a weakened defensive response to pain stimuli, compared to healthy controls.

In one of our previous studies [30], including 92 FM patients, we investigated the impact of depression and anxiety on cerebral pain processing in FM, not including any healthy controls. We found no difference in pain ratings or cerebral processing in patients with high depression and anxiety, suggesting that experimentally evoked pain in FM patients is not confounded by comorbid affective symptoms. The results from the present study are derived from the same patient cohort but concerns comparisons between FM patients and healthy controls, using connectivity analyses, which is different from any of our previous studies. However, our previous results may suggest that the present study is not confounded by patients’ affective symptoms.

### Thalamus connectivity

There is increasing evidence for altered thalamic function in pain patients with chronic pain such as neuropathic pain [58,59] and FM [9,23,24]. Previous literature suggests that lowered thalamic function in FM patients represents a ceiling effect of descending pain inhibition [9] maintained by the persistent excitatory input of pain signals. Support for this mechanism was found in a study where normalization of reduced thalamic activity was seen in response to analgesic treatment (nerve blockade) in patients with peripheral neuropathic pain [58]. When comparing the functional connectivity between FM patients and controls, using the thalamus as a seed, we found significantly higher connectivity between the thalamus and the lateral OBFC in healthy controls. This finding supports the general idea that chronic pain alters the thalamocortical connections, causing a disruption of thalamic feedback and the view of chronic pain as thalamocortical dysrhythmia [28].

The OBFC has been proposed to be involved in sensory integration, reward processing, decision-making and expectation [60]. A meta-analysis of previously published imaging data revealed that the medial part of the OBFC is related to reward whereas the lateral areas are more related to evaluation of noxious events and motivation to act upon these [61]. In a recent pain study, the OBFC was associated with reduction in pain unpleasantness after meditation training [62], indicating OBFC involvement in the evaluation of incoming pain signals. Moreover the lateral OBFC is central for placebo analgesia (compared to opioid analgesia) [42] and for anticipation of pain relief [63], furthering the notion that the OBFC plays an essential role in evaluation and inhibition of pain. However, there are also studies suggesting that the OBFC inhibits pain through neural projections that do not require conscious evaluation [64], arguing against the cognitive role of OBFC in pain inhibition. This alternate theory suggests that the OBFC is part of a functionally connected network that inhibits pain through homeostatic regulation of nociceptive input and endogenous inhibition [65]. Nevertheless, we believe that the reduced functional connectivity between the thalamus and OBFC in FM patients reflects lower function of pain regulation, possibly acting through a combination of cognitive evaluative processes and homeostatic control. A previous neuroimaging study by Guedj et al. [66], found that FM patients had lowered resting state perfusion of the prefrontal cortex, supporting our findings of impaired connectivity between crucial nodes of the pain inhibitory network of FM patients.

### Limitations

Due to the uneven number of FM patients and healthy controls available for this study, we chose to match each healthy control (n = 14) with two FM patients (n = 28). We estimate that the benefit of increasing our power by matching two patients per control, instead of one, is larger than the risk of getting unfair variance estimations for the two groups.

In the present study, we used subjectively calibrated pressure stimuli during fMRI scans, leading to matched levels of experienced pain in FM patients and controls. As a result, the pressure needed to evoke pain was significantly different for FM patients and controls and could possibly affect some aspects of our results. It is possible, but unlikely, that certain parts of the central nervous system can detect information about absolute stimulus magnitudes and thereby confound the comparison between FM patients and controls.

## Conclusion

In summary, our study indicates that FM patients display less functional connectivity between areas involved in pain inhibition compared to healthy controls during intermittent pressure pain administration. Our results suggest tha the dysfunction of the DPMS, a crucial homeostatic modulator of pain, plays an important role in maintenance of FM pain. These results may translate into clinical implications by using the functional connectivity of DPMS as an objective measure of pain dysregulation.

## Competing interests

The authors declare that they have no competing interests.

## Authors' contributions

EC, RHG, MI, EK, YM, FP, OV, SCRW participated in the design of the study. EC, SC, PF, MI, KBJ, EK, HM, YM FP, OV, SCRW coordinated the study and performed the data collection. Data analysis and expertise on analyses was contributed by PF, RLG, RHG, MI, KBJ, EK, JK, RL, HM, FP, SCRW. The manuscript was drafted by EC, SC, PF, RLG, RHG, MI, KBJ, EK, JK, RL, HM, FP, SCRW. All authors read and approved the final manuscript.
